# The United States College of Veterinary Surgeons

**Published:** 1897-09

**Authors:** 


### THE UNITED STATES COLLEGE OF VETERINARY SURGEONS.

The announcement for this year shows a change in the chair of dental surgery, Dr. William E. Yetton having succeeded Dr. Andrew C. Seacord in this department, and also on the hospital staff. The college has also very materially extended its list of qualifications for the degree of fellowship. In addition to the previous requirements, the applicant, if a graduate from a medical school, must have done some research-work in comparative medicine which has been of value to the veterinary world. He must submit a thesis on a new subject to have sufficient merit to be accepted by the college, and to be used by them in the compilation of a work on veterinary science. He is required to fill out an application blank, which will be furnished on application. The sum of ten dollars as an initiation fee will be charged, the money to be used in paying for the publication of these theses and the compilation of them into book-form, each Fellow of the college to receive a copy free of charge. This is done that the college may be able to compile a work that will be of great use to the profession, in that it hopes thereby to secure reports of practical experiments instead of theories which prove of but little consequence when applied to practical work.

